# Are university rankings useful to improve research? A systematic review

**DOI:** 10.1371/journal.pone.0193762

**Published:** 2018-03-07

**Authors:** Marlo M. Vernon, E. Andrew Balas, Shaher Momani

**Affiliations:** 1 Department of Clinical and Digital Health Sciences, College of Allied Health Sciences, Augusta University, Augusta, Georgia, United States of America; 2 Department of Mathematics, Faculty of Science, The University of Jordan, Amman, Jordan; 3 Nonlinear Analysis and Applied Mathematics (NAAM) Research Group, Faculty of Science, King Abdulaziz University, Jeddah, Kingdom of Saudi Arabia; Max Planck Society, GERMANY

## Abstract

**Introduction:**

Concerns about reproducibility and impact of research urge improvement initiatives. Current university ranking systems evaluate and compare universities on measures of academic and research performance. Although often useful for marketing purposes, the value of ranking systems when examining quality and outcomes is unclear. The purpose of this study was to evaluate usefulness of ranking systems and identify opportunities to support research quality and performance improvement.

**Methods:**

A systematic review of university ranking systems was conducted to investigate research performance and academic quality measures. Eligibility requirements included: inclusion of at least 100 doctoral granting institutions, be currently produced on an ongoing basis and include both global and US universities, publish rank calculation methodology in English and independently calculate ranks. Ranking systems must also include some measures of research outcomes. Indicators were abstracted and contrasted with basic quality improvement requirements. Exploration of aggregation methods, validity of research and academic quality indicators, and suitability for quality improvement within ranking systems were also conducted.

**Results:**

A total of 24 ranking systems were identified and 13 eligible ranking systems were evaluated. Six of the 13 rankings are 100% focused on research performance. For those reporting weighting, 76% of the total ranks are attributed to research indicators, with 24% attributed to academic or teaching quality. Seven systems rely on reputation surveys and/or faculty and alumni awards. Rankings influence academic choice yet research performance measures are the most weighted indicators. There are no generally accepted academic quality indicators in ranking systems.

**Discussion:**

No single ranking system provides a comprehensive evaluation of research and academic quality. Utilizing a combined approach of the Leiden, Thomson Reuters Most Innovative Universities, and the SCImago ranking systems may provide institutions with a more effective feedback for research improvement. Rankings which extensively rely on subjective reputation and “luxury” indicators, such as award winning faculty or alumni who are high ranking executives, are not well suited for academic or research performance improvement initiatives. Future efforts should better explore measurement of the university research performance through comprehensive and standardized indicators. This paper could serve as a general literature citation when one or more of university ranking systems are used in efforts to improve academic prominence and research performance.

## Introduction

Considering the significance of university innovation, there is a pressing need for outcome studies and quality improvement initiatives in the research enterprise. Keupp et al. [1] point out that current innovation management is characterized by conflicting predictions, knowledge gaps and theoretical inconsistencies. These issues may negatively impact the translation of academic research into discovery and applicable societal benefit. Research quality issues exist within university research; in the last 10 years, several studies and commentaries have highlighted the need for improvement in transparency, replicability, and meaningful research outcome reporting [2–6].

Many university administrators rely on university ranking systems as indicators of improvement over time and in comparison to other institutions. Universities promote improvement in standings as evidence of progress in the academic and research environments when requesting funding from government sources [7]. Other universities use ranking systems as evidence of cost-benefit for previously funded initiatives and to support additional funding requests. Consumers use university rankings to evaluate higher education opportunities both nationally and internationally.

Previous reviews of university rankings found that emphasis on reputation and institutional resources may not truly represent university quality [8–12]. Reviews of five ranking systems by Dill &Soo [8] focused on the suitability of rankings as representative of academic quality. Their findings demonstrate that ranking system indicators are not sufficient for promoting policy decisions or consumer choice. Suggested academic quality indicators include student entry criteria, program completion rates, proportion of graduates entering employment upon graduation, professional training, higher degrees, and the average starting salaries of graduates. Frey and Rost [13] concluded that publications and citations were not suitable indicators of scientific institutional worth. Their results suggest that multiple criteria should be implemented when assessing institutions for quality or choice for career decision.

Moed [12] most recently evaluated five world ranking systems and concluded that while ranking systems have improved in the last decade, the tendency to be one-dimensional hinders a more comprehensive university evaluation.

An evaluation of the Shanghai and Times Higher Education rankings conductd 70 simulations to replicate rankings; their results indicate that inaccurate weights were used to calculate the overall score [10]. The lack of replicability emphasizes the need for ongoing research quality evaluation and improvement. Trustworthiness of research influences not only scientific credibility but also effective innovation.

Assessment of the validity of research and academic quality indicators in university rankings is often unexplored; only once in the literature were two ranking systems so evaluated [14]. Integrating the much cited definitions of validity by Carmines and Hammersley, *validity* is the extent to which a measuring instrument accurately represents those features of a phenomena, that it is intended to describe[15,16].

While academic institutions have a responsibility to ensure that research process and outcomes efficiently and prudently manage resources, standardized research performance evaluation mechanisms for comparison across institutions do not currently exist. Academic institutions and administrators need reliable evaluation indicators of research and academic quality and university ranking systems are often used for this purpose. The objective of this study is to evaluate the usefulness of ranking systems for both academic and research performance and quality improvement, through a systematic review of publicly available university ranking systems.

## Methods

**Fig 1:**
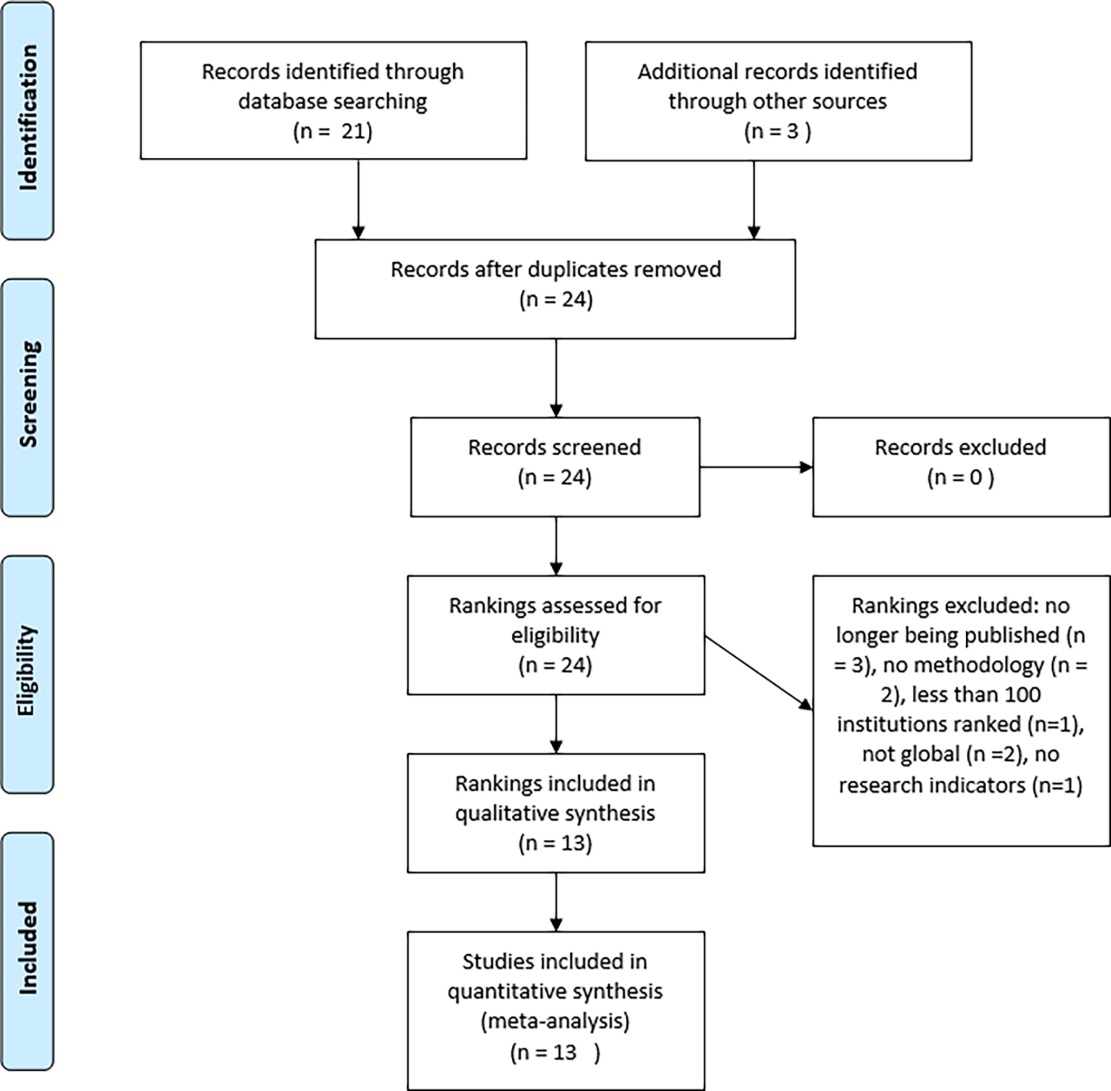
PRISMA flow diagram.

We conducted a systematic review of university ranking systems utilizing the PRISMA protocol and checklist, researched relevant measures to ascertain commonly used indicators for evaluating research performance and innovation (Fig 1, S1 Table) [17]. The review protocol for this study is available from the authors.

### Eligibility criteria

Ranking systems which include over 100 doctoral granting universities in their sample were eligible. Rankings must be currently produced on an ongoing basis and include US and global universities. Ranking systems also needed to publish rank calculation methodology in English. Ineligible criteria included rankings which were solely based on reputation surveys, did not include research outcome indicators or ranked institutions solely by subject area.

### Searches

A search of publicly available ranking systems for universities was undertaken between January and March 2017, through the use of internet search and qualitative literature review. Search terms included “university ranking”, “research productivity,” “measurement,” and “ranking university research.” Ranking system owners and VP of Research Administration were also consulted. Our searches were not limited to a certain field. Search engines used included PubMed (Search strategy: "university ranking"[All Fields]), Web of Science (WOS), and Google Scholar. To reduce selection bias, additional internet searches were also broadly conducted with the same search terms to identify any additional ranking systems.

### Processing/Abstraction

The purpose of the ranking system and methodologies for calculation of ranks were pulled from published statements through each ranking system website or publicly available documentation on methodology. Terms such as “the objective,” or “purpose of” each ranking system are used to identify the stated purpose of the ranking system. All indicators which were stated by the ranking systems to evaluate research and academics were abstracted and compared across systems. The aggregation methodology was also abstracted and compared from the publicly available methodologies and results.

### Analysis

Ranking systems were also evaluated on their utility for institutional quality improvement based on transparency of data and data analysis, consistency of indicators used in rankings over time, and availability of institution level data from ranking system–made available for others to replicate ranking calculations.

In this study, validity of ranking was assessed based on the following criteria: (i) content (i.e., comprehensiveness by including measures of both IP and publications, reliance on empirical data); (ii) consistency (i.e., transparency of indicator calculation; transparency of data/availability of raw institutional data; transparency of data aggregation; consistency of measures over time; process of ranking replicable); and (iii) resistance to bias (i.e., avoidance of self-reported data; does not rely on peer reputation surveys). Transparency of data is evaluated on the availability of raw institutional data used for comparison and whether the data can be used to analyze trends over time. The transparency of the data analysis algorithm is also evaluated as indicator transformations are provided with sufficient detail for replication and if the algorithms used for rankings are replicable by outside entities. The disclosure of the included percentage for each subscale used by the ranking system is included in this item. Subscales refer to the different components or indicators included in each ranking system’s overall score, for example, the percent of the overall score attributed to publications in high impact journals, total citations, or number of PhD graduates. To evaluate the appropriateness of rankings for use in research quality improvement action plans, the consistency of indicators over time is roughly assessed using a binary rating of present or not present. Consistency of indicators used over time is determined by publication of ranking methodology or indicator changes prior to rankings release, the stated frequency of changes, and whether included measures have a life cycle of inclusion. Resistance to bias of the ranking systems is assessed by whether or not data are self-reported to ranking systems, and the presence or absence of a stated validation process to confirm self-reported data is utilized by the system. Resistance to bias is also assessed by degree of reliance on empirical or qualitative survey data (majority percent of total score), as reputation surveys are not factors that institutions can control or design.

For the purposes of this study, the definition of *research performance* is based on standards for the NIH Research Performance Progress Report: publications, conference papers, and presentations; website(s) or other Internet site(s); technologies or techniques; inventions, patent applications, and/or licenses; other products, such as data or databases, physical collections, audio or video products, software, models, educational aids or curricula, instruments or equipment, research material, interventions (e.g., clinical or educational), or new business creation [18]. This review of university ranking systems looked for impact and products along these lines. Correspondingly, research performance indicators are interpreted as measures of publications, citations, and/or intellectual property.

*Academic quality* is defined as improvement in students' capabilities or knowledge as a consequence of their education at a particular college or university [19]. It is interpreted as measures pertaining to student progress or acheivement, and teaching quality as defined by faculty credintals.

## Results

A total of 24 ranking systems were initially identified through searches. Thirteen ranking systems which published in 2015 or 2016 were included in the results (Table 1). Excluded ranking systems were either no longer being published, did not include research performance indicators, or did not publish ranking methodologies. The range of institutions evaluated is between 500 and 5000 institutions. The oldest ranking system is the Carnegie Classification, established in 1973. All other ranking systems were first published between 2003 and 2015. Three ranking systems are run by universities, two by publications or news agencies, five by consulting or independent groups, and one by a government established entity. While the US News and World Report ranking of American universities was not eligible due to a lack of research performance indicators, the US News and World Report Global Ranking is included.

**Table 1:** University ranking systems.

Ranking System (abbreviation)	Initial Year	Sponsoring Organization	Total # of indicators	Frequency of publication	Participating Institutions	Version	Website
**Academic Ranking of World Universities (Shanghai)**	2003	Shanghai Ranking Consultancy	6	Annually	500	2016	http://www.shanghairanking.com/ARWU2016.html
**Carnegie Classification (Carnegie)**	1973	Carnegie Commission on Higher Education/ Indiana U.	8	Approximately every five years	4664	2015	http://carnegieclassifications.iu.edu/
**Center for World University Ranking (CWUR)**	2012	Center for World University Rankings	8	Annually	1000	2016	http://cwur.org/
**Leiden Ranking (Leiden)**	2011	Leiden University, Netherlands	18	Annually	842	2016	http://www.leidenranking.com/
**QS World University Ranking (QSWorld)**	2013	Quacquarelli Symonds Limited	6	Annually	916	2016	https://www.topuniversities.com/university-rankings
**Round University Ranking (RUR)**	2010	RUR Ranking Agency	20	Annually	761	2016	http://roundranking.com/
**SCImago Institutions Rankings World Report (SCImago)**	2009	SCImago Lab	12	Annually	5147	2016	http://www.scimagoir.com/
**The Times Higher Education World University Rankings (Times)**	2004	TES Global Ltd	13	Annually	800	2016	https://www.timeshighereducation.com/world-university-rankings
**Clarivate Analytics Innovative University Ranking (CA) (formerly Thomson Reuters)**	2015	Reuters	10	Annually	100	2016	http://www.reuters.com/article/amers-reuters-ranking-innovative-univers-idUSL2N1C406D
**U-Multirank (UMR)**	2014	European Union and Advisory Board	30	Annually	1200+	2016	http://www.umultirank.org/#!/home?name=null&trackType=home
**US News and World Report–Global Ranking (USN&W)**	2014	US News and World Report	12	Annually	1250	2016	https://www.usnews.com/education/best-global-universities/rankings
**University Ranking by Academic Performance (URAP)**	2010	Middle East Technical University	6	Annually	2000	2016	http://www.urapcenter.org/2016/
**Webometrics (Web)**	2004	Cybermetrics Lab, Spanish National Research Council	4	Biennial	11,995	2016	http://www.webometrics.info/en

The purpose of most ranking systems is to identify top institutions for consumers, to classify institutions by their research activity, and to compare institutions within countries and across the globe (Table 2). Some ranking systems state that they do not intend for the information to be used to compare institution to institution, but to provide a general interpretation of each institution’s annual performance.

**Table 2:** Stated purpose and use of ranking system.

Purpose	Ranking System
**Research Performance**	CWUR, Leiden, SCIMago, Times, RUR, Shanghai, URAP, UMR, Webometrics
**Research Quality**	Times, CA, UMR, URAP
**Research Innovation and Economic Impact**	CA
**University Comparison**	CWUR, QS World, RUR, Shanghai, Times, UMR, USN&WR
**University Marketing**	QS World, RUR, Shanghai, USN&WR
**Assist students in choosing an academic institution**	QS World RUR, Times, UMR, USN&WR
**Government funding or assessment**	RUR, Shanghai, Times, UMR
**Academic/Teaching Quality**	RUR
**Web Performance improvement**	Web

Four ranking systems specifically state that their results are intended to evaluate research quality. The Shanghai and UMR highlight their use in government cost benefit analysis; RUR, Shanghai, UMR, and Times state that their ranking systems may have use in supporting government funding requests.

The Carnegie Classification specifically states that their rankings are not intended to evaluate research performance. The Carnegie Classification System relies on R&D expenditure data in both STEM and non-STEM fields from the NSF Survey of Research and Development Expenditures at Universities and Colleges. Total staff working in science and engineering research are included from the NSF Survey of Graduate Students and Post-doctorates in Science and Engineering. No measures of research performance are assessed. The UMR system also provides indicators of quality, but leaves the definition of quality up to user preferences, by allowing a choice of indicators to be selected.

Tables 3 and 4 list the indicators utilized by the ranking systems to evaluate research performance or quality. Nine systems used total publications as an indicator–this is typically defined by the number of peer-reviewed articles that are included in either the Thomson Reuters Web of Science Core Collections database, or SCOPUS, produced by Elsevier. On average, 33.8% of ranking scores are assigned to publications and citations or various versions of these metrics. In most analyses, this is not dependent on first author affiliation, meaning that articles could be counted more than once across different institutions in collaborative works. Peer evaluation of both academic and research reputation and cumulative faculty awards contribute on average 39.8% of total ranking score among those who report weighting.

**Table 3:** Research indicators by publication and citation metrics (percent of contribution to total score, not all percentages may sum to 100, due to rounding.).

Metric	Data Sources	Carnegie	CWUR	Leiden	QSWorld	RUR	SCIMago	Shanghai	Times	CA	UMR	URAP	USN&WR	Web
**Number scientific documents (non-articles)**	WOS, Self-Reported										X	10%	5%	
**Number of Publications**	WOS, SCOPUS, SCI, InCites, Self-Reported		5%	X			8%	20%	6%	11.10%	X	21%	10%	
**Number of Citations (may be normalized)**	WOS, SCOPUS		5%	X	20%	8%	13%	20%	30%	11.10%	X	21%	7.50%	
**Number of Articles as Corresponding Author**	WOS						5%							
**Number of Articles in Nature or Science, or top 25% of journals**			5%				2%	20%						
**Number of Articles with External Collaboration**	WOS			X										
**Number of Articles with International Collaboration**	Scopus			X		4%	2%		2.50%		x	15%	10%	
**Number of Articles with Industry Collaboration**	WOS									11.10%	X			
**Number/Percent of Articles within the top most cited/field**	SCIMago Journal Rank indicator, WOS			x			2%				X	15%	32.50%	30%
**Number of Articles within the most cited as main contributor**	SCImago Journal Rank indicator, WOS						13%							
**Number of different authors from an institution**	SCOPUS						5%							
**Ratio of citations per publication**	InCites, WOS											18%	10%	
**Number of citations from top 10 producers at institution**	Google Scholar													10%
**Interdisciplinary of publications**											X			
**Ratio of Citations and Papers per staff**						16%								
**Industry Article Citation**	WOS									11.10%				
**H index of institution**	WOS		5%											

**Table 4:** Research indicators by intellectual property (percent of contribution to total score).

Metric	Data Sources	Carnegie	CWUR	Leiden	QSWorld	RUR	SCIMago	Shanghai	Times	CA	UMR	URAP	USN&WR	Web
**Patents Filed**	US PTO, WPO, DerWent World Patents Index, Derwent Innovations Index		5%							11.10%	X			
**Patents Awarded**	Derwent World Patents Index, Derwent Innovations Index, WPO									11.10%				
**Patents filed globally**	Derwent World Patents Index, Derwent Innovations Index									11.10%	X			
**Number of Publications cited in Patents applications**	PATSTAT, Patents Citation Index						30%			11.10%	X			
**Co-Patents with Industry**	PATSTAT										X			
**Start-Ups Initiated**	Self-Report										X			
**R&D Expenditures**	NSF, Self-Report	X							6%		X			
**R&D from Industry**	Self-Report								2.50%		X			
**Papers per research income**	Self-reported					2%								
**Research/Institutional income per staff and students**	Self-reported					6%								
**Science and Engineering (S&E) Staff**	NSF	X												
**Ratio of R&D to Institutional Income**	Self-Reported					2%								
**Non S&E R&D Staff**	NSF	X												
**Reputation Survey**	Independent Survey, Clarivate Analytics					8%			18%				25%	
**Total Faculty**	National Education Ministries, Self-report	X									X			
**Awards by Faculty/Alumni**	Nobel Prize, Fields Medal, others		50%					30%		11.10%				
**Summary Indicators by Faculty**	Ratio of weighted summary score by FTE of academic staff							10%						
**Total % focused on Research**		—	75%	100%	20%	46%	80%	100%	65%	100%	—	100%	100%	40%

Ranking systems which rely heavily on publication and citation metrics include the Leiden Ranking, Shanghai, SCImago, URAP, US News and World Report and the EU U-Multirank systems. The Leiden Ranking provides size-dependent and size-independent variants of all indicators, except publication output. Citation indicators are also normalized for scientific field differences. The counting method is conducted using a full counting and a fractional counting method- wherein collaborative publications are given less weight than non-collaborative ones (Leiden indicators description, page 4). An algorithm is applied to calculate field-normalized impact indicators, described by Waltman and Van Eck [20]. In the Shanghai ranking system, publications in Nature/Science and Nobel or Fields Awards comprise 50% of the score–indicating a reliance on highly selective indicators. Rankings are created by scoring the highest institution as 100, and the rest as a percentage of 100. URAP rankings are entirely based on publication and citation metrics. Scores are normalized according to field of study. CWUR rankings are the only ranking system that incorporates the *h*-index developed by Hirsch [21] to indicate the broad impact of a university’s research based on performance and citation impact. The *h-*index of an institution equals x if the institution has published x papers that have each been cited at least x times. For all but two ranking systems, Leiden and Carnegie, data used in the calculations are not made available making replicability of the rankings impossible. Leiden and Carnegie both provide downloadable spreadsheets of the ranking indicator data.

The percent of scores attributed to intellectual property (IP) measures, such as patents, was only 3.5% across all systems. Four systems incorporated at least one of these indicators–CWUR, SCImago, CA, and UMR. The Clarivate Analytics Most Innovative Universities is the only ranking system heavily focused on intellectual property indicators and includes indicators based on independent empirical data. A patent success ratio is calculated from patent awards per applications. Raw data is not available for validation and replication. The UMR, CWUR include patent applications. The one indicator of IP performance in SCImago is based on citation metrics (publications cited in patent applications) and heavily weights this in the summary score at 30%.

Academic quality indicators are presented in Table 5. Six systems incorporate academic quality by various indicators. The most common is a peer to peer survey, used by QS World, Times, US News and World Report, UMR, and RUR. Student/Faculty ratio is employed by each of these systems, excluding the US News and World Report. Carnegie, Times, and the UMR also use total doctoral degrees conferred when evaluating academic quality. Diversity of faculty and students are also used by QS World, Times, UMR and RUR as indicators of academic quality. CWUR attributes 25% of their ranking score to the number of alumni who are CEOs on the Forbes 100 list as the only measure of academic quality.

**Table 5:** Academic quality indicators table.

Academic		Carnegie	CWUR	Leiden	QSWorld	RUR	SCIMago	Shanghai	CA	Times	UMR	URAP	USN&WR	Web
**Reputation Survey–Academic Quality**	Independent Surveys, Student Survey				40%	8%				15%	X		X	
**Institutional Income**	Not specified									2.25%				
**Student/Faculty Ratio**	Not specified, self-reported				20%	8%				4.5%	X			
**Reputation Survey–Employer**	Independent 6urvey				10%									
**Doctoral Degrees Conferred**	IPEDS, Self-reported	X								2.25%	X			
**Doctoral Degree per admitted PhD candidate**	Self-Reported					8%								
**Doctoral Degree per Staff**	Self-Reported					8%								
**Faculty with Doctorates**	Self-Reported									6%	X			
**International Student Ratio**	Self-Reported				5%	2%				2.5%	X			
**International Faculty Ratio**	Self-Reported				5%	2%				2.5%	X			
**International students enrolled in Bachelor’s degree**	Self-Report					2%								
**Number of Alumni who are CEOs on Forbes 100**	Forbes Top 100 Companies		25%											
**Web presence**	Google						5%							10%
**Web domain inbound links**	Google						15%							50%
**Staff per Bachelor’s Degree**	Clarivate Analytics					8%								
**Doctoral degree per bachelor degree awarded**	Clarivate Analytics					8%								
**Total % Focused on Academics or Teaching Quality**		—	25%	0%	80%	54%	20%	0%	0%	35%	—	0%	0%	60%

The SCImago rank web presence by Google metrics makes up 20% of the total score. Similarly, Webometrics includes all global universities that have a web presence. The goal is to encourage universities and staff to increase their visibility through the number of webpages and external networks originating at institution websites. Citations and publications make up 40% of the score, based on the production of the most cited faculty.

Five ranking systems include reputation surveys as a significant component of the ranking calculation. The QS World ranking attributes 50% of the institution score to academic and employer reputation surveys. Research and academic reputation surveys contribute 33% of the Times ranking system.

An audit by PricewaterhouseCooper was completed for this methodology, yet there is no independent validation of self-report data or explanation of the weighting of the indicator percentages. Raw data is not provided for independent replication or validation. USN&WR Global Rankings incorporates surveys of global and regional research reputation (25% of the total score), the results of which are not publicly available. Round University Ranking, based out of Moscow, Russia, uses surveys for 16% of the overall score.

Standardization and aggregation methods are employed in various iterations by the ranking systems (Table 6). Efforts are made by all evaluated systems to normalize indicators by calculating ratios according to faculty numbers or research expenditures. Others normalized citations by field of study to lessen advantage of highly cited disciplines. Z scores, fractional counting, and weighted subscales are also used to standardize the ranking scores.

**Table 6:** Standardization and aggregation of indicators.

Method of Aggregation	Explanation	Ranking System
**Weighted per capita**	Data is standardized by ratio of total faculty or is weighted by discipline	Carnegie, QS World, RUR, SCIMago, Shanghai, Times, UMR
**Scores are normalized to produce rankings**	Scores are normalized to rank between 0 and 100	Carnegie, CWUR, QS World, RUR, SCIMago, Shanghai, CA, USN&WR, Web
**Raw data normalized by year and discipline**	Raw data is normalized by percentages according to field of study or year of publication	Leiden, Times, UMR, URAP, USN&WR, Web,
**Cumulative Probability Function using Z-scores**	Subscales are normalized using Z-scores	CWUR, Times, UN&WR
**Cumulative scores are percentages of subscales**	Subscales are assigned percentages when calculating the total scale	CWUR, QS World, RUR, SCImago, Shanghai, CA, Times, URAP, Web
**Fractional counting method**	Collaborative data is weighted by ratio of total authors’ participating institutions	Leiden, Shanghai
**Rank Groups**	Classification into groups based on the distance of the indicator score from the median or group mean	Carnegie, UMR

The suitability of ranking systems for use in research performance improvement is reported in Table 7. It provides a rough binary assessment of the various ranking systems on the different dimensions. All ranking systems refine their analysis prior to each publication. No ranking systems report any specific measures or analysis of their indicator validity. Leiden provides a stability interval to support the individual indicator.

**Table 7:** Suitability of ranking for research performance improvement.

	Carnegie	CWUR	Leiden	QSWorld	RUR	SCIMago	Shanghai	Times	CA	UMR	URAP	USN&WR	Web
**Comprehensive (includes measures of both IP and publications)**	-	-	-	-	-	X	-	-	X	X	-	-	-
**Reliability Measures**	-	-	-	-	-	-	-	-	-	-	-	-	-
**Transparency of Indicator Calculation Methodology**	X	X	X	-	X	-	-	X	X	-	X	-	-
**Transparency of Data/Availability of raw institutional data**	X	-	X	-	-	-	-	-	-	-	-	-	-
**Transparency of Data Aggregation**		-	X	-	X	X	-	-	X	-	X	-	-
**Consistency of Measures Over Time**	-	-	X	-	X	-	X	-	-	X	-	-	-
**Process of Ranking Replicable**	X	X	X	-	-	-	X	-	X	-		-	-
**Avoidance of Self-Reported Data**	X	-	X	X		X	X		X	-	X	X	X
**Reliance on Empirical data**	X	X	X	-	-	X	-	-	X	X	X	-	X
**Does not rely on Peer Reputation Surveys**	X	X	X	-	-	X	X	-	X	-	X	-	X

One research institution was compared across all ranking systems in Table 8, to demonstrate the variability of ranking systems.

**Table 8:** Conflicting global rankings of an illustrative research university (per most recent published results, 2016).

Ranking System	Actual Rank	Relative Rank (% of total)
**Carnegie Classification**	Highest Research Activity	n/a
**Center for World University Ranking**	50	5.2
Leiden Ranking*	n/a	n/a
**QS World University Ranking**	71	7.7
**Round University Ranking**	66	8.6
**SCImago Institutions Rankings World Report**	60	2.1
**Academic Ranking of World Universities/Shanghai**	93	18.6
**The Times Higher Education Supplement**	52	4.9
**Thomson Reuters Innovative University Ranking**	24	24.0
**University Ranking by Academic Performance**	125	6.2
U-Multirank*	n/a	n/a
**US News and World Report–Global Ranking**	66	6.6
**Webometrics**	40	0.03

* There is no overall rank.

## Discussion

Administrators, funders, and consumers should look for rankings which are consistent over time, cover multiple areas of measurement and are less reliant on peer reputation. Based on our results, reputation surveys, self-reported and unvalidated data, and non-replicable analyses create an impractical foundation for research improvement assessment, and can lead to a wide range of institutional ranks. When rankings are used to as support for budget requests, or as evidence of return on investment, indicators which provide a balanced approach have the best opportunity to be truly reflective.

When used in tandem, several ranking systems may have more reasonable comprehensiveness and validity. Use of the Leiden Ranking System, the Clarivate Analytics Innovation Ranking System, and SCImago process for systematic evaluation and comparison may be a promising approach for research administrators. The U-Multirank is the broadest of the systems examined, but without the ability to compare a university’s performance over time rather than in overall categories, trend analysis becomes difficult.

We found that current ranking systems rarely incorporate the promotion of innovation culture through patents or intellectual property disclosures. Increasing the research product: publication/patent, may be easily manipulated to increase rankings without actually increasing contribution to science [22,23].

In our sample, eight of the thirteen systems include indicators to measure academic quality. These are mainly focused on peer reputation, faculty achievement, student to faculty ratios, and the total number of awarded doctorates in both STEM and non-STEM fields. Valid measures of academic quality are not universally standardized [8]. Many ranking systems are marketed either for academic choice/comparison, yet, these indicators do not sufficiently reflect the teaching and learning environments of students.

Research expenditure is often used an indicator of the strength and quality of an institution’s research capabilities. However, no correlation has been found between more research expenditure and better quality research. A Canadian evaluation found a diminishing rate of return between the two factors, and in the US, NIH funding was significantly correlated with increased publications, but not with development of novel therapeutics [24,25].

University rankings tend to focus on bibliometric sources which are biased towards English language journals and are therefore not comprehensive or fully accurate. Peer reputation surveys are not published, nor is the data made available, and bias towards larger more well-known institutions may be inevitable. In addition, measures such as the number of Nobel Prize winners could be considered “luxury” indicators, accessible to elite universities but are out of reach and un-motivating for most other universities.

In this review, we explore the validity and suitability of ranking systems for research performance improvement. Clearly, there is a need for improvement in ranking methodologies. Applying organizational management principles may improve the validity and reliability of university ranking systems and assist with appropriate indicator choices.

We propose that the ideal ranking systems limits the significance of peer reputation to no more than 10%, and meets the Comprehensiveness, Transparency and Replicability criteria described in Table 5. Current approaches rely on easily accessible output data sources; reliance on these measures perpetuates the perspective that a few approaches adequately represent scientific value, quality improvement and innovation performance. While we believe this represents a comprehensive analysis of appropriate ranking systems, other institutions may rely on different systems. Consultation with ranking system developers and research administrators has provided support for the included list.

## Conclusions

There is a need for a credible quality improvement movement in research that develops new measures, and is useful for institutions to evaluate and improve performance and societal value. Quality over quantity should be emphasized to affirm research performance improvement initiatives and outcomes, which benefit society through scientific discovery, economic outcomes, and public health impact. Current indicators are inadequate to accurately evaluate research outcomes and should be supplemented and expanded to meet standardized criteria. We suggest that future research evaluate three dimensions of research outcomes: scientific impact, economic outcomes, and public health impact for evaluating research performance within an academic institutional environment.

## Supporting information

S1 Table PRISMA checklist. (DOC)
